# Negative affective burden is associated with higher resting-state functional connectivity in subjective cognitive decline

**DOI:** 10.1038/s41598-022-10179-y

**Published:** 2022-04-13

**Authors:** Claudia Schwarz, Gloria S. Benson, Daria Antonenko, Nora Horn, Theresa Köbe, Olga Klimecki, Werner Sommer, Miranka Wirth, Agnes Flöel

**Affiliations:** 1 Department of Neurology, Charité – Universitätsmedizin Berlin, Corporate Member of Freie Universität Berlin and Humboldt-Universität zu Berlin, Berlin, Germany; 2 NeuroCure Clinical Research Center, Charité – Universitätsmedizin Berlin, Corporate member of Freie Universität Berlin and Humboldt-Universität zu Berlin, Berlin, Germany; 3 Institute for Molecular Medicine Finland (FIMM), HiLIFE, University of Helsinki, Helsinki, Finland; 4 Department of Geriatric Psychiatry, Central Institute of Mental Health, Medical Faculty Mannheim, Heidelberg University, Mannheim, Germany; 5 Department of Neurology, University Medicine Greifswald, Greifswald, Germany; 6 German Centre for Neurodegenerative Diseases, DZNE, Dresden, Germany; 7 Psychology Department, Technische Universität Dresden, Dresden, Germany; 8 Department of Psychology, Humboldt-Universität zu Berlin, Berlin, Germany; 9 Department of Psychology, Zhejiang Normal University, Jin Hua, China; 10 German Center for Neurodegenerative Diseases (DZNE), Rostock/Greifswald, Greifswald, Germany

**Keywords:** Cognitive ageing, Cognitive neuroscience, Alzheimer's disease

## Abstract

Subjective cognitive decline (SCD), as expressed by older adults, is associated with negative affect, which, in turn, is a likely risk factor for Alzheimer’s Disease (AD). This study assessed the associations between negative affective burden, cognitive functioning, and functional connectivity in networks vulnerable to AD in the context of SCD. Older participants (60–90 years) with SCD (n = 51) and healthy controls (n = 50) were investigated in a cross-sectional study. Subclinical negative affective burden, quantified through a composite of self-reported negative affective factors, was related to cognitive functioning (self-perceived and objective) and functional connectivity. Seed-to-voxel analyses were carried out in default mode network (DMN) and salience network (SAL) nodes using resting-state functional magnetic resonance imaging. Greater negative affective burden was associated with lower self-perceived cognitive functioning and lower between-network functional connectivity of DMN and SAL nodes in the total sample. In addition, there was a significant moderation of SCD status. Greater negative affective burden related to higher functional connectivity within DMN (posterior cingulate-to-precuneus) and within SAL (anterior cingulate-to-insula) nodes in the SCD group, whereas in controls the inverse association was found. We show that negative affective burden is associated with functional brain alterations in older adults, regardless of SCD status. Specifically in the SCD phenotype, greater negative affective burden relates to higher functional connectivity within brain networks vulnerable to AD. Our findings imply that negative affective burden should be considered a potentially modifiable target for early intervention.

## Introduction

Subjective cognitive decline (SCD) in older adults denotes the expression of perceived cognitive difficulties and associated worry in the absence of objective cognitive impairment. As a potential risk factor for developing Alzheimer’s Disease (AD)^1–3^, SCD has been linked to biomarkers of neuropathology^4–6^ and increased risk of clinical progression^7,8^. A central aspect of SCD is the presence of negative affective factors, as characterized by subclinical anxiety and depression as well as repetitive negative thinking and neuroticism^9–11^. Negative psycho-affective factors have been associated with brain abnormalities as well as increased risk of cognitive decline and clinical progression^12–16^ and are considered potentially modifiable risk factors of age-related cognitive decline and AD development in older adults^14,16^.

In this light, furthering knowledge about the impact and relevance of negative affective burden in the context of SCD is important in order to facilitate the development of early intervention strategies^17^. Recent work proposes a central role of negative affect in depleting resilience against brain pathology and accelerating cognitive decline in aging individuals^18,19^. It is specifically proposed that psychological risk factors tap a common construct of negative affect, covering a range of negative mood states including depression, worry, stress coping, rumination and RNT. According to this concept of ‘cognitive debt’^18^, negative affect and maladaptive responses to stressors may constitute a psychological risk profile that potentially increases risk for developing AD. In a major recent advance, existing studies provided first evidence for the relationship between negative affective factors with neuroimaging markers of AD pathology and/or subsequent cognitive decline^20,21^.

Altered functional connectivity in resting-state brain networks are known to presage clinical manifestation of AD, and, in the setting of SCD^22–24^, are considered a sensitive indicator for early brain abnormalities^25,26^. Evidence further suggests that affective factors may explain functional alterations within key brain networks vulnerable to AD. Two main networks, derived from resting-state functional magnetic resonance imaging (fMRI), are particularly relevant to AD-related affective changes: the default mode network (DMN), supporting self-referential processing, and the salience network (SAL), implied in socio-emotional processing and emotion regulation^27,28^. Notably, greater negative affective burden has been associated with higher functional connectivity for core nodes of the SAL or the DMN in patients with mild AD^29^ as well as in cognitively unimpaired older adults^30^, but not patients with mild cognitive impairment (MCI)^31^. Moreover, higher functional connectivity within the DMN is associated with late-life depression in older adults^32^. Overall, it appears that negative affective burden may relate to aberrant functional connectivity in brain networks affected by AD with increased functional connectivity in regions pertaining higher-order cognitive and emotional processing. However, to our information, none of the existing studies focused on the relationship between negative affective burden and functional connectivity in the context of SCD.

While negative affective burden helps form the behavioral signature of SCD, a comprehensive characterization of the psycho-affective profile and its association with cognitive and brain functioning remains to be accomplished. Therefore, the current study investigated the associations between subclinical negative affective burden, cognitive functioning (objective and self-perceived), and functional connectivity related to those brain networks that show early abnormalities in AD, namely the DMN and the SAL. Here, we operationalized negative affective burden as a composite, encompassing several self-reported negative psycho-affective measures including subclinical depression, rumination, negative stress coping and neuroticism. We then examined the association of negative affective burden with cognitive behaviors and functional brain connectivity among older adults with SCD and among healthy controls (HC). Based on the literature, we expected that greater negative affective burden would be associated with lower cognitive functioning (objective and self-perceived), particularly in those participants at greater risk of AD. In addition, we explored the associations between negative affective burden and functional connectivity for selected DMN and SAL nodes in older adults with SCD as compared to controls. Previous studies imply that greater negative affective burden may relate to higher functional connectivity in the DMN and SAL. The nature of these associations in the context of SCD is currently unknown.

## Methods

### Participants

The data used in this study were obtained from the SmartAge SCD study (ClinicalTrials.gov: NCT03094546) data release (June 1st, 2018). Further details of the study protocol are provided elsewhere^33^. At the time of analysis, data from 101 participants, 51 individuals with SCD and 50 HC were available. The SCD group was part of the SmartAge randomized trial, while the HC group was recruited with a similar baseline protocol. Screening and baseline assessments were performed on the same day.

For the present study, only cognitively unimpaired individuals (60–90 years old) were recruited. The presence of SCD was diagnosed using established guidelines^1^ that included presence of cognitive complaints for at least 6 months, self-reported worry related to the cognitive complaints, and endorsement to seek medical help due to these complaints. Older adults were included as HC participants, if they did not report subjective cognitive worsening or if they were not concerned about subjective cognitive worsening. All participants had to pass an on-site screening ensuring normal cognitive performance. Details of the on-site screening and exclusion criteria have been published previously^33^.

All participants underwent baseline assessment of psycho-affective behavior, self-perceived and objective cognitive functioning, and functional magnetic resonance imaging. One participant was excluded from data analysis due to drop-out and four participants were excluded from imaging analysis due to missing magnetic resonance imaging (MRI) data (n = 3) and failed quality control procedures (n = 1, for details see below). Therefore, 100 participants (50 SCD, 50 HC) were included for behavioral analysis and 96 participants were included for imaging analysis (49 SCD, 47 HC).

The study was conducted in accordance with the declaration of Helsinki and approved by the ethics committee of the Charité—University Medicine Berlin Germany (EA1/250/16). All participants provided informed written consent and received reimbursement.

### Cognitive measures

A battery of neuropsychological tests assessed cognitive performance^33^ from which a global cognitive measure was derived similar to the Preclinical Alzheimer’s Cognitive composite (PACC). The measure was designed to provide distinctive information about early signs of AD-related cognitive decline in still cognitively unimpaired individuals^34^. To create composite scores the following individual test scores were z-transformed and averaged: total Mini-Mental State Examination (MMSE) score, total immediate learning recall from the German version of the Auditory Verbal Learning Test (AVLT)^35^, the logical memory total delayed recall, and the Digit Symbol substitution test^36^.

### Behavioral measures

Self-reported questionnaires were administered on site and at home. Psycho-affective measures were selected from the questionnaire battery described in detail elsewhere^33^. Subclinical depressive affect was assessed through the Geriatric Depression Scale (GDS) short version (15 items). Rumination was assessed with the 23-item Response Style Questionnaire (RSQ-D), which quantifies the extent to which individuals respond to negative events by focusing on self, symptoms, and distraction^37^. Stress coping was evaluated with the 78-item Stress Coping Style Questionnaire (SVF-78), which assesses the particular coping style (positive or negative) of an individual with stressful situations^38^. Neuroticism, a personality trait, was assessed using the Big-Five Inventory (BFI) neuroticism score from the 10-item questionnaire^39^.

Self-perceived cognitive functioning was assessed using the 39-item Everyday Cognition Scale (ECog-39) measuring subjective cognitive changes in multiple domains in comparison to ten years ago. The global score was calculated as the total amount of affirmatively answered questions across the 39 items, reported in the established scoring method^40^, with higher scores indicating lower self-perceived cognitive functioning.

### Assessment of negative affective burden

Motivated by the conceptual framework proposed by Marchant and Howard^18^, we evaluated negative affective burden as a composite score over psychological scales related to negative affect. Psycho-affective measures were selected to best represent the individual’s psychological risk. We conducted an exploratory principal component analysis (PCA) followed by varimax rotation^41^, to explore the relationship among the psycho-affective measures. The Kaiser–Meyer–Olkin (KMO) measure verified the sampling adequacy for the analysis, KMO = 0.70, and Bartlett's test of sphericity *X*^2^ (28) = 253.411, *p* < 0.01, indicated that correlations between items were sufficiently large for PCA. Two components had eigenvalues above Kaiser's criterion of 1 and together explained 59.8% of the variance. Factor loadings after rotation are shown in Table 1.

**Table 1:** Summary of principal component analysis to extract the negative affective burden composite for the entire sample.

Psycho-affective measures	Rotated factor loadings	
	PC1: negative affective burden	PC2: psychological resilience
Subclinical depression	**0.633**	−0.373
Symptom-rumination	**0.880**	0.073
Self-rumination	**0.758**	0.357
Neuroticism	**0.616**	−0.508
Negative coping	**0.831**	−0.134
Distraction	0.109	**0.804**
Positive coping	−0.119	**0.820**

Factor loadings pertaining to a factor appear in bold.

*PC* principal component.

The first principal component (PC1) was composed of subclinical depression, symptom- and self-rumination, negative coping and neuroticism, suggesting that this component represents negative affective burden. The second component (PC2) with positive coping and distraction reflected psychological resilience related to positive coping strategies. Given the specific aim of the study, the resulting factor scores from PC1, thereafter referred to as negative affective burden composite, was extracted and used for further analysis with higher scores indicating greater negative affective load.

### MRI acquisition

Anatomical brain scans were acquired using a 3 Tesla Siemens scanner (Tim Trio, Siemens, Erlangen, Germany) at the Berlin Center for Advanced Neuroimaging (BCAN, Charité – Universitätsmedizin Berlin). T1-weighted images were acquired using a magnetization-prepared rapid acquisition gradient-echo (MPRAGE) sequence with the following parameters: repetition time (TR) = 1900 ms; echo time (TE) = 2.52 ms; 192 sagittal slices; size = 1.0 × 1.0 × 1.0 mm^3^; flip angle = 8°. Functional scans were obtained at rest using a T2*-weighted echo-planar imaging (EPI) sequence TR = 2300 ms; TE = 30 ms; 34 axial slices acquired in interleaved order; size = 3.0 × 3.0 × 4.0 mm^3^; flip angle = 90°. During the 7-min resting-state scan participants were instructed to keep their eyes closed and not think of anything in particular.

### Preprocessing of resting-state fMRI

The publicly available CONN Functional Connectivity Toolbox version 17c (http://www.nitrc.org/projects/conn), which is based on SPM12 (Wellcome Department of Cognitive Neurology, London, UK; www.fil.ion.ucl.ac.uk/spm) was used to analyze resting-state fMRI data^42^. Default preprocessing steps including functional realignment, unwarping, slice-time correction, outlier detection, functional normalization, structural segmentation and normalization to the Montreal Neurological Institute (MNI) template were used. In addition, functional images were smoothed using an 8-mm Gaussian kernel. Confounds in the blood oxygenation level-dependent (BOLD) signal from physiological and other spurious sources of noise were estimated and regressed out using the anatomical component-based noise correction (CompCor) method^43^ as implemented in CONN (scrubbing, motion regression, and filtering [0.008–0.09 Hz]). Anatomical images were segmented into gray matter (GM), white matter (WM) and cerebrospinal fluid (CSF) for the removal of temporal confounding factors (WM and CSF). The identification of outlier scans was performed using artifact detection toolbox.

Next, we used the established seed-to-voxel analytical approach implemented in the CONN toolbox to derive individual first-level within-subject connectivity maps. A similar method was chosen in our previous studies^44,45^. First-level whole-brain correlational maps were generated for each participant by extracting the mean resting-state BOLD time course for each seed and calculating Fisher’s r-to-z-transformed correlation coefficients with the BOLD time course throughout the whole brain. For each functional network, we used the ‘atlas’ regions-of-interest (ROIs) from the DMN and SAL as seed regions, namely the posterior cingulate cortex (PCC; center of gravity in MNI coordinates: 1, −61, 28; size: 38,664 mm^3^) for the DMN (see also Pistoia and colleagues^46^) and the anterior cingulate cortex (ACC; center of gravity in MNI coordinates: 0, 22, 35; size: 8,504 mm^3^) for the SAL. The selected ROIs are provided in the CONN toolbox and were derived from an independent component analysis of the human connectome project dataset (497 participants). The BOLD signal was averaged within each atlas-based ROI. As done in our previous work^44^, we chose these seeds, given that they are characterized as core network nodes and both showed early abnormities in AD development^27,29,47^. Individual connectivity maps were then subjected to voxel-wise second-level analysis.

### Additional measures

Additional variables comprising risk factors for AD were assessed for the demographic characterization of the diagnostic groups (SCD and HC). Educational attainment was measured in years of education. Self-reported family history of AD and non-specified dementia were defined within first-degree family members. Genotype information on apolipoprotein E (APOE) ε4 status was obtained, using whole blood samples taken from all participants, as described in a previous report^4^.

### Statistical analyses

Statistical analyses were performed with SPSS software v24.0 (PASW, SPSS; IBM, Armonk, NY) and R v3.5.0 (available online at https://www.R-project.org/). Differences between diagnostic groups (SCD, HC) were assessed on selected demographic, cognitive, and behavioral measures using independent *t*-test for continuous variables and Chi-square test for categorical variables.

#### Behavioral analysis

At first, associations between the negative affective burden composite and behavioral measures were examined. Separate linear regression analyses were conducted to investigate associations of negative affective burden (independent variable) with both objective as well as self-perceived cognitive functioning (dependent variables) in the total sample, adjusted for SCD group status. Subsequently, the interaction between negative affective burden and SCD group status was investigated, by adding the interactive term (SCD group status × negative affective burden) to each statistical model. Our confirmatory behavioral analyses were corrected for the multiple analyses performed using a two-sided significance level of α = 0.025.

#### Functional connectivity analysis

We examined the associations between the negative affective burden composite and resting-state functional connectivity in the pre-selected resting-state brain networks (DMN and SAL). To this aim, separate whole-brain second-level linear regression analyses were carried out in the total sample with the negative affective burden composite as independent variable and the individual voxel-wise first-level connectivity maps as dependent variables, as previously done^44,48^, adjusted for SCD group status. Subsequently, the interaction between negative affective burden and SCD group status was investigated at the voxel level, by adding the interactive term (SCD group status × negative affective burden) to the statistical model carried out in CONN^42^.

As stated in the introduction, none of the existing studies have assessed the association between negative affective burden and functional connectivity in the context of SCD. Therefore, it was our goal to obtain deeper understanding and generate further hypotheses on these associations based on our data. This was done by choosing a rather exploratory approach^49^. Our functional connectivity analyses were not corrected for the seeds (PCC and ACC) tested in our study, due to the exploratory approach; thus, we applied a two-sided significance level of α = 0.05 for the cluster-extent threshold, as described further below. As in our previous studies^44,45^, the statistical parametric maps obtained for each network seed were thresholded using a joint cluster-forming height threshold of *p* < 0.005 (uncorrected) and extent threshold of *p* < 0.05 corrected for multiple comparisons using false discovery rate (FDR)^50^ to account for the large number of comparisons in a seed-to-voxel analysis. The usage of *p* < 0.005 (uncorrected) for the voxelwise threshold is lenient but acceptable in order to detect smaller effects^51^. Notably, it has been argued that at single study level a greater Type I error rate is acceptable to avoid Type II errors^51^. Average individual functional connectivity values were extracted from the significant clusters as z-scores and used as functional connectivity measures for visualization. All associations were plotted for visual representation and interpretation of directionality, using the R package Jtool (https://cran.r-project.org/web/packages/jtools/).

## Results

### Demographic data

Demographic data are shown in Table 2. Participants in both groups did not differ significantly in terms of age, education, sex ratio, APOE status or global cognitive performance (all *p’s* ≥ 0.108). The SCD group involved a higher percentage of individuals indicating a family history of dementia and, as expected, reported significantly lower self-perceived cognitive functioning compared to controls, but did not differ in objective cognitive performance. Individuals with SCD showed higher preclinical depression, self- and symptom-rumination, negative stress coping, and neuroticism scores compared to the HC group (all *p’s* ≤ 0.035), while groups did not differ in distraction and positive stress coping (all *p’s* ≥ 0.379). Correspondingly, the negative affective burden composite was higher in individuals with SCD than in controls (*p* < 0.001).

**Table 2:** Demographics and sample characteristics.

	HC (n = 50)	SCD (n = 50)	*p*
**Demographics**			
Women—n (%)	25 (50)	24 (48)	0.841
Age (years)	71 (6), 60 to 85	70 (6), 60 to 83	0.200
Education (years)	16 (3), 10 to 29	16 (4), 11 to 27	0.920
Family history of dementia—n (%)	7 (14)	26 (52)	< 0.001
APOE ε4 carrier—n (%)	7 (14)	10 (20)	0.451
**Objective cognition (scores)**			
PACC (composite score)	0.10 (0.63), −1.30 to 1.51	−0.12 (0.72), −2.21 to 1.34	0.108
**Self-perceived cognition (scores)**			
Global score^a^	1.3 (0.3), 1.0 to 2.4	1.8 (0.5), 1.1 to 3.6	< 0.001
**Psycho-affective measures (scores)**			
Subclinical depression^b^	0.8 (0.8), 0 to 3	1.9 (1.7), 0 to 6	< 0.001
Self-rumination^c^	10.6 (2.8), 7 to19	11.9 (3.1), 7 to 18	0.035
Symptom-rumination^c^	11.4 (3.0), 8 to 20	13.8 (3.9), 8 to 24	< 0.001
Distraction^c^	18.9 (5.4), 8 to 28	19.7 (4.2), 9 to 32	0.379
Stress coping positive^d^	13.5 (3.1), 6.6 to 21.1	13.1 (2.4), 6.9 to 18.0	0.440
Stress coping negative^d^	8.3 (3.3), 0.8 to 16.8	10.1 (4.4), 2.3 to 21.0	0.027
Neuroticism^e^	2.6 (0.9), 1 to 5	3.1 (1.0), 1 to 5	0.021
Negative affective burden (composite score)	−0.4 (0.8), −1.9 to 1.9	0.4 (1.1), −1.7 to 2.7	< 0.001

If applicable, measures are expressed as mean (standard deviation) and range. APOE ε4 carrier status of one participant of the healthy controls (HC) group is missing.

*APOE ε4* apolipoprotein E ε4, *PACC* Preclinical Alzheimer’s Cognitive Composite, *SCD* subjective cognitive decline.

^a^Everyday Cognition Scale (ECog).

^b^Geriatric Depression Scale (GDS).

^c^Response Style Questionnaire (RSQ).

^d^Stress Coping Style Questionnaire (SVF-78).

^e^Big-Five Inventory (BFI).

### Relationship between negative affective burden and behavioral measures

Results from the linear regression analyses, evaluating the associations between the negative affective burden composite and behavioral measures, are presented in Table 3. Greater negative affective burden was significantly associated with lower self-perceived global cognitive functioning (*β* = 0.491*,* CI 0.335 to 0.647,* p* < 0.001) regardless of SCD group. No association was found with global cognitive performance (*β* = −0.006, CI −0.219 to 0.208, *p* = 0.959). The SCD group status showed no significant moderation effect on these associations, since the interactions between diagnostic group and the negative affective burden composite were not significant for the dependent variables of objective cognitive functioning (*β* = 0.008, CI −0.354 to 0.370, *p* = 0.965) and self-perceived cognitive functioning (*β* = 0.006, CI: −0.259 to 0.272, *p* = 0.963).

**Table 3:** Associations between negative affective burden and SCD status on objective and self-perceived cognitive functioning from the linear regression analysis.

Independent variables	Objective cognition			Self-perceived cognition^#^		
	Un. B	β	*p*	Un. B	β	*p*
**a. Main effect**						
Negative affective burden	−0.004	−0.006	0.959	0.223	0.491	< 0.001
SCD group status	−0.218	−0.160	0.140	0.307	0.340	< 0.001
**b. Interaction effect**						
SCD group status × negative affective burden	0.007	0.008	0.965	0.004	0.006	0.963
R^2^	0.026			0.477		

Group coded: SCD = 1, HC = 0. Un. B are unstandardized B values, β indicate standardized B values, R^2^ represents the variance of the data which is explained by the model.

*HC* healthy controls, *SCD* subjective cognitive decline.

^#^Higher scores proportionally relate to lower self-perceived cognitive functioning.

### Relationship between negative affective burden and functional connectivity

**Figure 1:**
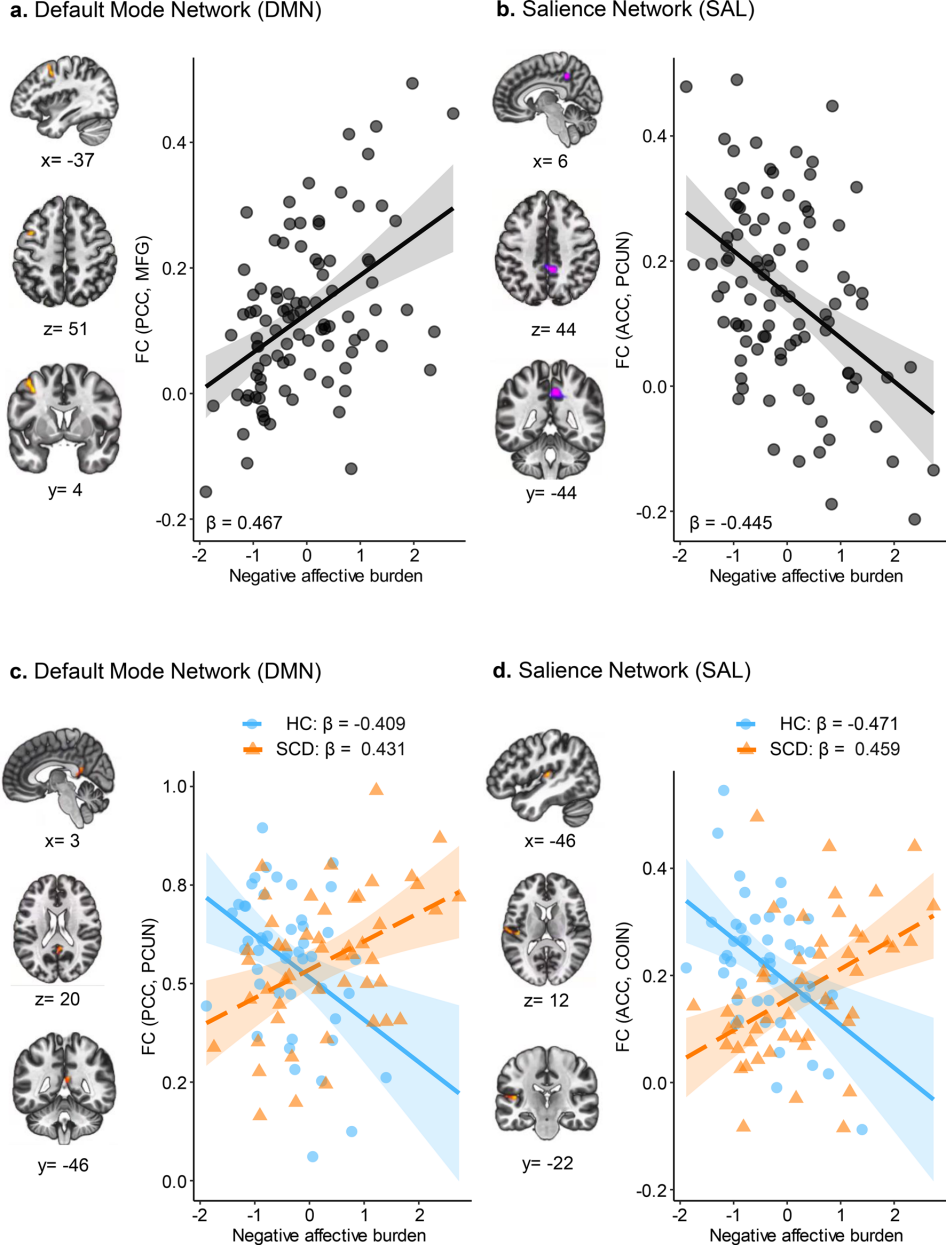
Effect of negative affective burden on functional connectivity. (**a**,**b**) Main effects of the negative affective burden composite on functional connectivity. For the Default Mode Network (**a**, PCC seed), greater negative affective burden was associated with higher coupling between the PCC and the left middle frontal gyrus (MFG). For the Salience Network (**b**, ACC seed), greater negative affective burden was associated with lower functional connectivity between the ACC and the precuneus (PCUN). (**c**,**d**) Interactions between the negative affective burden composite and subjective cognitive decline (SCD) group status on functional connectivity. Greater negative affective burden was associated with higher regional functional connectivity in the SCD group and with lower regional functional connectivity in the healthy control (HC) group. For the Default Mode Network (**c**, PCC seed), brain regions included the cingulate gyrus/PCUN and for the Salience Network (**d**, ACC seed) the left central opercular/insular cortex (COIN). (**a**–**d**) Maps were displayed with a voxel-level threshold of *p* < 0.005 and a cluster-level threshold of *p* < 0.05 FDR-corrected. Individual relationships were illustrated using scatterplots with fitted regression, shaded areas indicate 95% confidence intervals and dots represent individual functional connectivity values extracted from significant clusters of the voxel-wise regression analyses. Standardized beta values (β) are presented for main (**a**,**b**) and interaction effects (**c**,**d**). *ACC* anterior cingulate cortex, *FC* functional connectivity, *FDR* false discovery rate, *HC* healthy controls, *PCC* posterior cingulate cortex, *SCD* subjective cognitive decline.

Resting-state whole-brain functional connectivity was analyzed with the PCC and ACC as seed regions of the DMN and SAL networks, respectively. Results of the main effect seed-to-voxel analyses, evaluating the association between the negative affective burden composite and local functional connectivity in the total sample (n = 96) are shown in Fig. 1a,b with cluster information presented in Table 4a. For the DMN, greater negative affective burden was significantly related to higher functional connectivity between the PCC (seed region) and the left middle frontal gyrus (MFG) (*β* = 0.467, CI: 0.286 to 0.648). For the SAL, greater negative affective burden was associated with lower functional connectivity between the ACC (seed region) and the precuneus (*β* = −0.445, CI −0.628 to −0.262).

**Table 4:** Associations between negative affective burden and SCD status on functional connectivity.

Anatomical regions	Number of voxel	*p*
**a. Main effect: Negative affective burden**		
Default mode network (seed: PCC)		
Middle frontal gyrus, left	200	0.023
Salience network (seed: ACC)		
Precuneus	342	0.031
**b. Interaction effect: SCD group status × negative affective burden**		
Default mode network (seed: PCC)		
Precuneus/cingulate gyrus	134	0.020
Salience network (seed: ACC)		
Central opercular/insular cortex, left	181	0.019

Results of the seed-to-voxel regression analysis are presented at cluster level for the main effect (A) and interactive effect (B) between negative affective burden and subjective cognitive decline (SCD) status. Statistical parametric maps were thresholded using a height threshold of *p* < 0.005 (uncorrected) at the voxel level and an extend threshold of *p* < 0.05 (FDR-corrected) at the cluster level. For each significant cluster, this tables specifies the corresponding anatomical region, the number of voxels (actual cluster size) and the FDR-corrected *p* value.

*ACC* anterior cingulate cortex, *FDR* false discovery rate, *PCC* posterior cingulate cortex.

Next, we investigated a potential moderation of SCD group status on the functional associations, as assessed by the interaction between the negative affective burden composite and SCD group status at the voxel level. A significant interaction between SCD group status and negative affective burden was found for the DMN node, namely the PCC to the precuneus (*β* = 0.776, CI: 0.419 to 1.134), and for the SAL node, namely the ACC to the left central opercular/insular cortex (*β* = 0.836, CI: 0.493 to 1.179). Visual inspection of this significant interaction showed that greater negative affective burden was associated with increased functional connectivity between the PCC-to-precuneus and the ACC-to-insular cortex in the SCD group (PCC-to-precuneus: *β* = 0.431, CI: 0.166 to 0.696; ACC-to-insular: *β* = 0.459, CI: 0.198 to 0.720). In contrast, in the HC group, greater negative affective burden was associated with lower functional connectivity between the PCC-to-precuneus and the ACC-to-insular cortex (PCC-to-precuneus: *β* = −0.409, CI −0.683 to −0.136; ACC-to-insular: *β* = −0.471, CI −0.736 to −0.207). Results of the seed-to-voxel analyses with an interaction are shown in Fig. 1c,d with cluster information provided in Table 4b.

## Discussion

The present findings demonstrate the relevance of negative affective burden in older adults with and without SCD. Across participants of both diagnostic groups (SCD, HC), greater negative affective burden was associated with lower self-perceived cognitive functioning, but not with lower objective cognition. In the total sample, greater negative affective burden correlated with altered between-network functional connectivity of DMN (posterior cingulate-to-middle frontal) SAL (anterior cingulate-to-insula) nodes. Moreover, we observed a moderation of SCD status. Greater negative affective burden was associated with higher functional connectivity within DMN (posterior cingulate-to-precuneus) and within SAL (anterior cingulate-to-insula) nodes in the SCD group, whereas in controls the inverse association was found. Overall, our findings corroborate the importance of considering negative affective burden in cognitive and brain aging and as a potentially modifiable target for early intervention.

In general, our results confirm that negative affective burden is correlated with self-perceived cognitive complaints in older adults. More precisely, greater negative affective burden, here assessed using a composite of subclinical depression, rumination, negative coping and neuroticism, was associated with lower self-perceived cognitive functioning in the total sample. This relationship mirrors existing findings, supporting a reliable link between lower psychological well-being and self-perceived cognitive difficulties in older adults^52^. In line with cross-sectional findings^16,53^ and a synthesized review^54^, the observed behavioral association was found regardless of whether or not participants were diagnosed with SCD using established diagnostic guidelines^1^. The result thus proposes a more general interrelation between psychological wellbeing and self-perceived cognitive health in the older population. Contrary to our expectation, we did not find a significant association between our negative affective burden composite and objective cognitive functioning, as evaluated with a sensitive cognitive risk marker for AD^34^. Although negative affective factors have been related to increased risk for cognitive decline over time^12,13,20^, similar associations may not be present in baseline behavioral responses of healthy older adults.

As an important novel finding, we show a moderation of the SCD status on the association between negative affective burden and functional connectivity within the DMN and SAL networks. More precisely, greater negative affective burden was associated with higher functional connectivity within DMN (PCC-to-precuneus) and within SAL (ACC-to-insular cortex) nodes – only in the SCD group. The inverse association was observed in controls, where greater negative affective burden correlated with lower functional connectivity within the respective brain networks. In both networks, the DMN and SAL, regional functional connectivity alterations have been associated with SCD in older adults^22,24,55^. The current study extends this evidence and identifies a distinct neural correlate of negative affective burden in the SCD phenotype compared to controls, resembling the patterns described in previous studies. For example, our results echo reports, showing that higher functional coupling within DMN nodes is related to less positive emotion in older adults, regardless of amyloid status^30^. Moreover, higher functional connectivity within SAL nodes has been related to neuropsychiatric burden in mild AD^29^. Finally, increased functional connectivity within DMN nodes is reported in “high ruminators”^56^ as well as in older adults with late-life depression^32^, where this pattern is thought to reflect abnormalities in self-referential mental processes as individuals fixate on negative thoughts^56^.

Our results imply that the neural signature of negative affective burden is distinguishable in older adults with SCD compared to controls. This interesting finding was generated using an exploratory approach. Yet, our observation is important, given the evidence of neuropathology^57,58^ and AD-typical patterns of brain injury^5,59–61^ in those individuals at greater risk for developing AD^8^. While the pathological burden found in SCD may facilitate early neural disruptions, the exact implications of the known functional connectivity alterations^22,24,55^ remain up for debate. A recent reconciliatory review of the functional neuroimaging findings proposes a model, whereas higher functional connectivity is viewed as a key characteristic of early SCD and may reflect information processing inefficiencies in the DMN, SAL, and executive control networks^26^. Higher functional connectivity within DMN and within SAL nodes, as associated with negative affective burden, may thus constitute a neural correlate of the affective and emotional dysregulation thought to be present in older adults with SCD^62^. By contrast, in the controls greater negative affective burden was associated with lower functional connectivity within DMN and within SAL nodes. These negative associations were unexpected and could not be inferred from prior studies^29–31^. One might speculate that lower intra-network functional connectivity could perhaps correspond to a neural downregulation in the setting of greater negative affect, as previously demonstrated in healthy non-depressed adults^63^. In general, this finding could reflect the presence of differential regulatory processes in older adults with SCD compared to controls in functional brain networks vulnerable to AD.

We further demonstrate a more general association between negative affective burden and altered brain functioning in older adults. Greater negative affective burden was correlated with altered functional connectivity of the DMN and SAL nodes to other higher-order brain regions in the entire sample. Although heterogeneous in directionality, the aberrant functional coupling could reflect a neural signature of self-referential negative affective states, such as rumination and worry, thought to comprise large-scale brain networks^64^. Interestingly, the here-observed pattern involves the three inter-related neurocognitive networks that also play a major role in cognitive and affective disorders^17,65^. We found that greater negative affective burden was associated with higher functional connectivity between the PCC seed (DMN) and the MFG. The latter region is a key node of the executive control network^66^, hinting towards an intensified engagement of attentional resources^67^ in the self-referential processes. At the same time, greater negative affective burden was associated with lower functional connectivity between the ACC seed (SAL) and the precuneus, as a key node of the DMN. This pattern, as similarly seen in rumination, is suggested to reflect an aberrant interplay between the two brain networks subserving salience attribution and internal mentation, respectively, and may contribute to a disturbed allocation of cognitive resources^68^. Taken together, our findings propose that negative affective burden (at subclinical level) could be associated with altered between-network functional connectivity, irrespective of the SCD status. The observation may reflect a remodeling (or “wear-and-tear”^69^) in the coordination of the large-scale brain circuitry implicated in a wide range of cognitive and emotional processes^65^.

In sum, our study corroborates important links between a psychological signature of negative affective burden, self-perceived cognitive changes, and altered brain functioning in older adults. Through its impact on brain functioning, negative affective burden is proposed to increase ‘cognitive debt’, thereby presumably aggravating vulnerability towards AD^18^. Negative affective states become more frequent in early or preclinical stages of cognitive dysfunction^70^ and can be related to patterns of AD pathology^20,58^. Negative affective burden may thus be an important and potentially modifiable risk factor for AD development^12–14^. The ongoing identification of the underlying neurophysiological mechanisms serving negative affective burden may help characterize those individuals most susceptible to further decline. Those at-risk due to negative affect burden might respond to targeted behavioral interventions, such as cognitive therapy^71^ or mindfulness-based training^72^, as preventive strategies. Overall, the current study highlights the need for further clarification of the impact of negative affective burden on healthy and pathological aging. More research on the neural signature of negative affective burden is required to better understand possible implications for the development of AD and to identify effective intervention strategies. To disentangle temporal relationships between negative affective burden, altered brain functioning, and cognitive failure longitudinal studies are needed, particularly in older adults with SCD.

There are some important limitations to consider. First, our study is limited by its cross-sectional approach. Thus, the observed associations of negative affective burden do not permit causal interpretation. Further longitudinal studies are needed to capture the value and trajectory of functional connectivity alterations as subjective decline becomes objective. Second, our measure of negative affective burden composite was derived from our sample and needs further validation. The study of psycho-affective measures as risk factors is heterogeneous in the field, with similar proxies (i.e., “neuropsychiatric burden”, “affective symptoms”) converging comparable results^54^. On the other hand, our well-described data driven and evidence-based construction of the psychological composite, may facilitate the use of this construct in future SCD studies. Some might argue that certain negative affective factors, like depression, should be treated as confounding factors, however, the guidelines in the field of SCD suggest otherwise as it provides an incomplete understanding of the expression of SCD^1,2^. Lastly, we used an exploratory approach in our functional connectivity analysis, which is a limitation in terms of the generalization of our findings to the general population. The present functional connectivity findings were also restricted to two pre-selected resting-state networks in agreement with our a-priory assumptions. The current results on the association between negative affective burden and aberrant functional connectivity in older adults with and without SCD should be validated by studies in independent cohorts that may incorporate other measures of inter- and intra-network connectivity along with mediation models (e.g., using the clusters we observed as pre-defined ROIs).

In conclusion, our results demonstrate the functional relevance of negative affective burden in older adults, where it is associated with altered self-perceived cognitive functioning and functional brain connectivity irrespective of SCD status. In the SCD phenotype, greater negative affective burden relates to higher functional connectivity within brain networks that are vulnerable to AD, whereas in controls the inverse association is observed. Our findings imply that negative affect may be a worthy target of early intervention.

## Data Availability

The data that support the findings of this study are available in anonymized form from the corresponding authors on reasonable request.
